# Enzymatic and structural properties of human glutamine:fructose-6-phosphate amidotransferase 2 (hGFAT2)

**DOI:** 10.1074/jbc.RA120.015189

**Published:** 2020-12-17

**Authors:** Isadora A. Oliveira, Diego Allonso, Tácio V.A. Fernandes, Daniela M.S. Lucena, Gustavo T. Ventura, Wagner Barbosa Dias, Ronaldo S. Mohana-Borges, Pedro G. Pascutti, Adriane R. Todeschini

**Affiliations:** 1 Laboratório de Glicobiologia Estrutural e Funcional, Instituto de Biofísica Carlos Chagas Filho (IBCCF), Universidade Federal do Rio de Janeiro (UFRJ), Rio de Janeiro, RJ, Brazil; 2 Departamento de Biotecnologia Farmacêutica, Faculdade de Farmácia, UFRJ, Rio de Janeiro, RJ, Brazil; 3 Laboratório de Modelagem e Dinâmica Molecular, IBCCF, UFRJ, Rio de Janeiro, RJ, Brazil; 4 Laboratório de Macromoléculas, Diretoria de Metrologia Aplicada às Ciências da Vida, Instituto Nacional de Metrologia, Qualidade e Tecnologia (INMETRO), Duque de Caxias, RJ, Brazil; 5 Laboratório de Genômica Estrutural, IBCCF, UFRJ, Rio de Janeiro, RJ, Brazil

**Keywords:** glutamine:fructose-6-phosphate amidotransferase (GFAT), glucosamine-6-phosphate synthase, hexosamine biosynthetic pathway (HBP), carbohydrate metabolism, enzyme kinetics, protein structure, molecular modeling, molecular dynamics, αKG, α-ketoglutaric acid, APAD, 3-acetylpyridine adenine dinucleotide, APADH, reduced form of APAD, EGS, ethylene glycol bis(succinimidyl succinate), Fru-6P, fructose-6-phosphate, G6PD, glucose-6-phosphate dehydrogenase, GDH, glutamic acid dehydrogenase, GFA, glutamine:fructose-6-phosphate amidotransferase from *C. albicans*, GFAT, glutamine:fructose-6-phosphate amidotransferase, Glc-6P, glucose-6-phosphate, GlcN-6P, glucosamine-6-phosphate, GlmS, glucosamine-6-phosphate synthase from *E. coli*, GLN, glutaminase (in reference to protein domain or activity), HBP, hexosamine biosynthetic pathway, hGFAT, human GFAT, IPTG, isopropyl-β-D-thiogalactoside, ISOM, isomerase (in reference to protein domain or activity, LC-MS, liquid chromatography coupled to mass spectrometry, MD, molecular dynamics, mGFAT, murine GFAT, PGI, phosphoglucose isomerase, rhGFAT, recombinant human GFAT, RMSF, root mean square fluctuation, TOCSY, total correlation spectroscopy

## Abstract

Glycoconjugates play a central role in several cellular processes, and alteration in their composition is associated with numerous human pathologies. Substrates for cellular glycosylation are synthesized in the hexosamine biosynthetic pathway, which is controlled by the glutamine:fructose-6-phosphate amidotransfera-se (GFAT). Human isoform 2 GFAT (hGFAT2) has been implicated in diabetes and cancer; however, there is no information about structural and enzymatic properties of this enzyme. Here, we report a successful expression and purification of a catalytically active recombinant hGFAT2 (rhGFAT2) in *Escherichia coli* cells fused or not to a HisTag at the C-terminal end. Our enzyme kinetics data suggest that hGFAT2 does not follow the expected ordered bi–bi mechanism, and performs the glucosamine-6-phosphate synthesis much more slowly than previously reported for other GFATs. In addition, hGFAT2 is able to isomerize fructose-6-phosphate into glucose-6-phosphate even in the presence of equimolar amounts of glutamine, which results in unproductive glutamine hydrolysis. Structural analysis of a three-dimensional model of rhGFAT2, corroborated by circular dichroism data, indicated the presence of a partially structured loop in the glutaminase domain, whose sequence is present in eukaryotic enzymes but absent in the *E. coli* homolog. Molecular dynamics simulations suggest that this loop is the most flexible portion of the protein and plays a key role on conformational states of hGFAT2. Thus, our study provides the first comprehensive set of data on the structure, kinetics, and mechanics of hGFAT2, which will certainly contribute to further studies on the (patho)physiology of hGFAT2.

Glycoconjugates are particularly diverse in structure and composition and play a central role in several cellular processes such as cell growth, cell–cell and cell–matrix adhesion, cell differentiation, among others. Severe alterations in the composition of glycoconjugates are usually associated to human diseases (1, 2). The primary substrates for intra- and extracellular glycosylation are obtained through the hexosamine biosynthetic pathway (HBP), which is controlled by the rate-limiting enzyme glutamine:fructose-6-phosphate amidotransferase (GFAT) (3).

The enzyme GFAT belongs to the amidotransferase family, class II, characterized by an N-terminal cysteine as the nucleophilic catalyst (4). All cellular organisms including prokaryotes and eukaryotes express this class of enzymes, highlighting their relevance to normal cell functioning. Indeed, deletion of the GFAT gene in *Escherichia coli* and *Saccharomyces pombe* led to cell death (5). In mammals, GFAT was characterized in 1960 in rat liver homogenates, when Ghosh *et al.* (6) described its specificity for fructose-6-phosphate (Fru-6P) and not for glucose-6-phosphate (Glc-6P) to generate glucosamine-6-phosphate (GlcN-6P). In humans, three different isoforms of GFAT were reported, named hGFAT1, hGFAT1Alt (or GFAT1-L), and hGFAT2, encoded by the *gfpt1* and *gfpt2* genes, respectively. hGFAT1 expression is ubiquitous, and it is highly expressed in the placenta, pancreas, and testis (7). hGFAT1Alt represents an expanded isoform of hGFAT1 resulting from alternative splicing of the *gfpt1* gene, and its expression is restricted to striated muscle (8, 9). In turn, hGFAT2 is the product of a distinct gene, *gfpt2*, and shares 79% identity with hGFAT1 (7). hGFAT2 presents a more restricted expression pattern than hGFAT1, being the major isoform in several central nervous system tissues and observed in a smaller proportion in the heart, placenta, testis, and ovary (7).

Interest in hGFATs has increased in the past few years as this protein has been implicated in human pathologies. The hGFATs play a direct role in type 2 diabetes, and their overexpression contributes to insulin resistance and higher *O*-GlcNAc levels (10, 11, 12). In fact, previous work has identified hGFATs as potential targets for the development of antidiabetes drugs (12, 13). In addition to diabetes, hGFAT has been assigned a prominent role in the close relationship between HBP and cancer. hGFAT1 isoform has been observed to be upregulated in breast (14), prostate (15), and hepatic (16) cancers. On the other hand, it has been observed that hGFAT2 levels increased considerably in pancreatic adenocarcinoma (17) and colorectal cancer (18).

Despite its importance in cellular metabolism, there are few studies of the biochemical and kinetics properties of mammalian GFATs. The structure of GFAT is characterized by having two domains, glutaminase (GLN) and isomerase (ISOM), responsible for its enzymatic activity. Its complete reaction mechanism was proposed from studies with GlcN-6P synthase (GlmS), the bacterial homolog of hGFAT, being characterized as bi–bi-ordered in which the entry of Fru-6P induces conformational changes that favor glutamine (Gln) binding (19). Concerning the hGFATs, most studies to date have focused on unraveling the mechanisms and structure of isoform 1 (20, 21, 22). This isoform naturally occurs as a homotetramer, which is its active oligomeric state (21, 23). In contrast to *E. coli* GlmS, there were few crystal structures of the hGFAT1 isomerase (ISOM) domain, and only very recently the full structure of this isoform was reported (22). Conversely, there is only one report focusing on the expression, purification, and kinetics of the recombinant variation of the murine GFAT2 (mGFAT2) (24).

Here, we explore hGFAT2 biochemical properties, reporting its low catalytic efficiency and providing evidences, which indicates that its enzymatic mechanism is different from the bacterial one.

## Results

### Recombinant human GFAT2 (rhGFAT2) forms tetramers in solution

We expressed the recombinant hGFAT2 (rhGFAT2) protein in *E. coli* cells with and without a 6xHisTag at its C-terminal end. The best expression condition for both constructs was achieved after 0.5 mM IPTG induction for 6 h at 25 °C under agitation. Although the majority of rhGFAT2 was expressed as inclusion bodies, a small fraction remained soluble (Fig. S1, *A*–*B*). To avoid improper refolding, we purified rhGFAT2 from the soluble fraction in a Ni^+2^NTA column, and we obtained highly pure (96% purity) HisTag-containing rhGFAT2 (rhGFAT2-his) protein after a single step of affinity chromatography (Fig. S1*A*, Table 1). Surprisingly, rhGFAT2 without HisTag (rhGFAT2 w/o tag) also bound to Ni^+2^NTA column, comprising therefore the first step for its purification, which reached high purity level (96%) after an additional step of an anion exchange chromatography in a Q-sepharose column (Fig. S1*B*, Table 1). Despite the high purity of both samples, final yield of purified rhGFAT2 w/o tag was 10 times lower than that of rhGFAT2-his (Table 1). The absence of the HisTag was further confirmed by western blot analysis using an anti-HisTag monoclonal antibody (Fig. S1*C*).

**Table 1:** Purification of rhGFAT2s in *E. coli*

Purified protein	Specific activity^a^ (U/mg)	Yield^b^ (mg GFAT/l growth culture)	Purity (%)
rhGFAT-his	2.7 × 10^−4^	13.4	96
rhGFAT2 w/o tag	2.8 × 10^−4^	1.7	96

a Enzyme units activity is defined as the specific activity was expressed as units (μmol of GlcN-6P synthesized per min at 37 °C) per mg of protein.

b Protein concentrations were determined by the method of Bradford using bovine serum albumin as standards.

To assess whether purified enzymes were functional, we performed an enzymatic assay to detect the GlcN-6P formation. As shown in Table 1, both rhGFAT2-his and rhGFAT2 w/o tag exhibited similar specific activity (Table 1), suggesting that HisTag at the C-terminal end has little or no effect on enzyme function. Based on these results and the overall yield of purified enzymes, we further used rhGFAT2-his in biochemical characterization studies.

**Figure 1:**
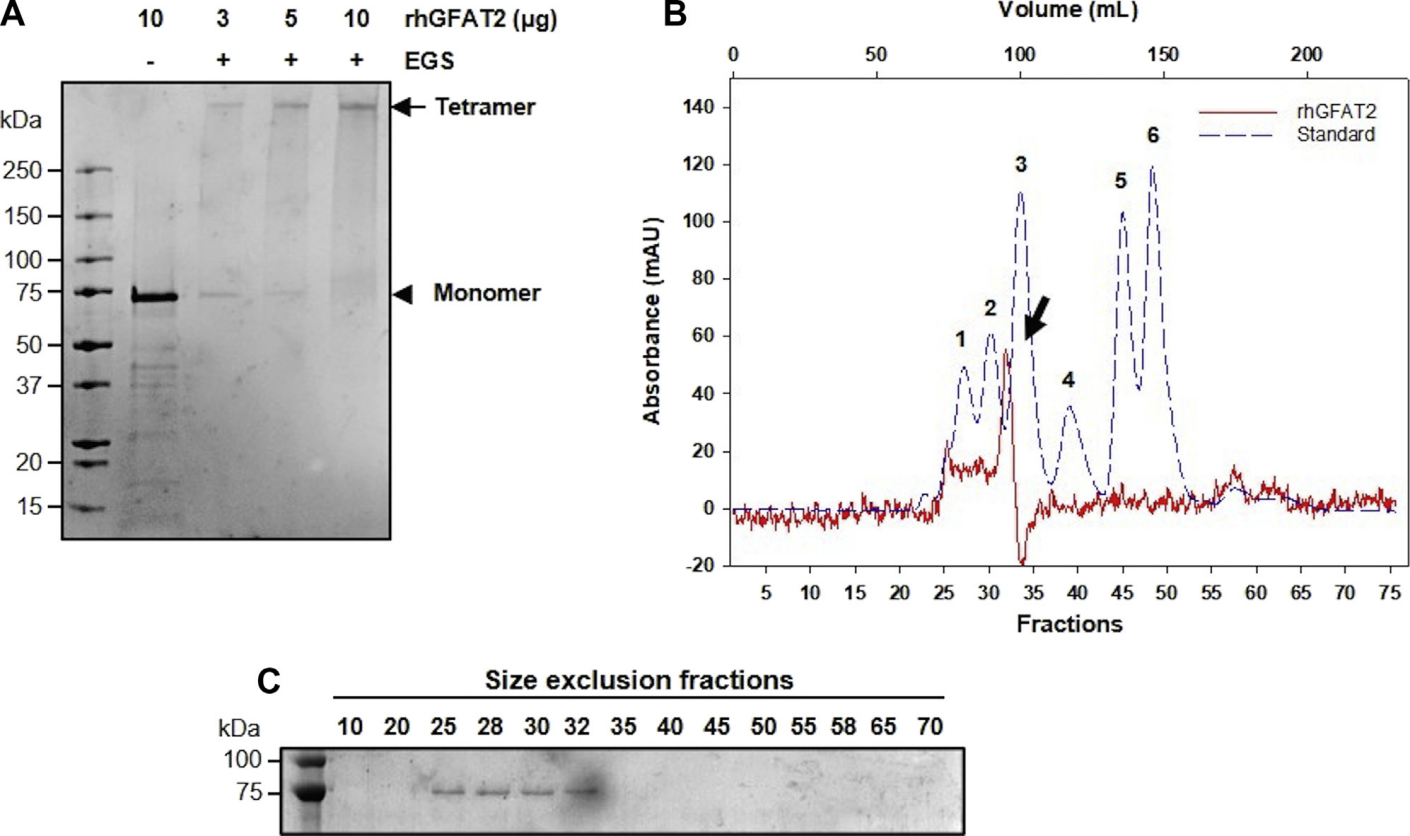
**Evaluation of the rhGFAT2 oligomeric state.***A*, cross-linking assay in which 3, 5, or 10 μg of hGFAT2-his was incubated in the presence of 1 mM EGS. The control was performed by incubation of 10 μg of rhGFAT2-his in the absence of EGS. *Arrow* and *arrowhead* represent the tetramer and monomer, respectively. *B*, size-exclusion chromatogram of rhGFAT2-his (*solid red line*) in Superdex 200 16/200 column. The *arrow* represents the major peak of the enzyme. Standard proteins (*dashed blue line*) were subjected to the same condition as rhGFAT2 and are described as follows: 1—Thyroglobulin (669 kDa), 2—Apoferritin (443 kDa), 3—β-amylase (200 kDa), 4—BSA (66 kDa), 5—Carbon anhydrase (29 kDa), and 6—Cytochrome c oxidase (12.4 kDa). The collected fractions were subjected to SDS-PAGE followed by Coomassie blue staining (*C*).

As the oligomeric states of hGFAT1 seem important to its activity, we performed a cross-link assay using ethylene glycol bis(succinimidyl succinate) (EGS) and different amounts of rhGFAT2. We observed the presence of oligomers larger than 250 kDa in all conditions analyzed (Fig. 1*A*). To confirm this finding and ascertain the protein multimeric form, we performed a size-exclusion chromatography using a Superdex 200 column. The rhGFAT2-his was mostly eluted at the retention volume of 95 ml (Fig. 1, *B*–*C*), which refers to molecular weight of approximately 300 kDa, consistent with the expected weight of the rhGFAT2-his tetramer. We also observed an additional peak at approximately 74 ml followed by a shoulder up to 92 ml, consistent to multiple oligomeric forms, up to octamers (Fig. 1, *B*–*C*). Together, our results demonstrate that rhGFAT2 can be successfully expressed in *E. coli* cells and the purified protein forms tetramers in solution.

### Enzyme kinetics of rhGFAT2

To have a detailed perspective on hGFAT2 kinetics, we measured its GlcN-6P synthetic activity (Fig. S2*A*) using a modified Elson–Morgan reaction (25, 26) and the ability of each domain to hydrolyze Gln or isomerize Fru-6P through specific coupled assays. All kinetic parameters are summarized in Table 2. As GlcN-6P synthetic activity of hGFAT is supposed to follow a bisubstrate ordered mechanism based on kinetic studies of *E. coli* GlmS (19, 27), we initially used that mechanistic model equation to fit our rate *versus* substrate curves (Fig. S2*A*). However, we obtained an inconsistent (negative) value for ^*Fru-6P*^*K*_*M*_. Hence, we used Michaelis–Menten model to obtain apparent values of *K*_*M*_ and *k*_*cat*_. The ^app^*K*_*M*_ value for Fru-6P is higher than for Gln (0.957 and 0.763 mM, respectively) (Table 2), and rhGFAT2-his exhibited low ^app^*k*_*cat*_ value for GlcN-6P synthesis (Table 2).

**Table 2:** Kinetic parameters of reactions catalyzed by rhGFAT2-his

Type of activity	Substrate(s)	*K*_*m*_ (Gln) (mM)	*K*_*m*_ (Fru-6P) (mM)	*k*_*cat*_ (min^−1^)
Aminohydrolyzing activity	w/o Fru-6P	0.820 ± 0.335	–	0.021 ± 0.003
	w/Fru-6P	1.814 ± 1.158	–	0.079 ± 0.016
Isomerase activity	w/o Gln	–	0.711 ± 0.170	0.322 ± 0.022
GlcN-6P synthetic activity	Fru-6P	–	0.957 ± 0.502^a^	0.032 ± 0.007^a^
	Gln	0.763 ± 0.332^a^	–	0.040 ± 0.008^a^

a The kinetic parameters for synthase activity were generated through Michaelis–Menten fitting, therefore must be considered as apparent values.

It is known that the first Met residue is removed during heterologous protein expression (28, 29), and it was also reported for GFAT (22, 30). The role of Cys2 as GFAT N-terminal catalytic residue (27, 31) corroborates the need for Met1 removal. Therefore, as the presence of HisTag did not affect the overall synthetic activity (Table 1), we further assessed whether the N-terminal sequence of rhGFAT2-his was intact. The peptide fingerprint suggests that Met1 was properly removed from rhGFAT2-his, as observed in the coverage of the detected peptides (Fig. S3*A*), which was confirmed by the fragmentation pattern of the N-terminal peptide 2-CGIFAYMNYRVPR-14 (Fig. S3*B*). Thus, the reduced activity of rhGFAT2-his cannot be explained by alteration in primary protein sequence.

We then monitored the release of glutamic acid during Gln hydrolysis. We observed that hGFAT2-his is able to hydrolyze Gln even in the absence of Fru-6P, but the presence of this phosphorylated monosaccharide increases four times the *k*_*cat*_ of aminohydrolysis reaction (Table 2). The kinetic curves show a large increase in the Gln hydrolysis’ rate promoted by Fru-6P (Fig. S2*B*). Despite the *k*_*cat*_ of aminohydrolyzing activity being in the same order of magnitude of synthetic activity (around 0.03 min^−1^), the isomerase activity exhibits a 10-time higher *k*_*cat*_ (0.322 min^−1^), and a *K*_*M*_ of 0.711 mM for the sugar (Table 2). In contrast to aminohydrolysis, the analysis of the isomerase activity curves in the presence of increasing concentrations of Gln indicates that hGFAT2-his performs the isomerization of Fru-6P to Glc-6P even in high concentrations of this amino acid (Fig. S2*C*), suggesting that part of the ammonia released from Gln hydrolysis is not used for GlcN-6P synthesis.

**Figure 2:**
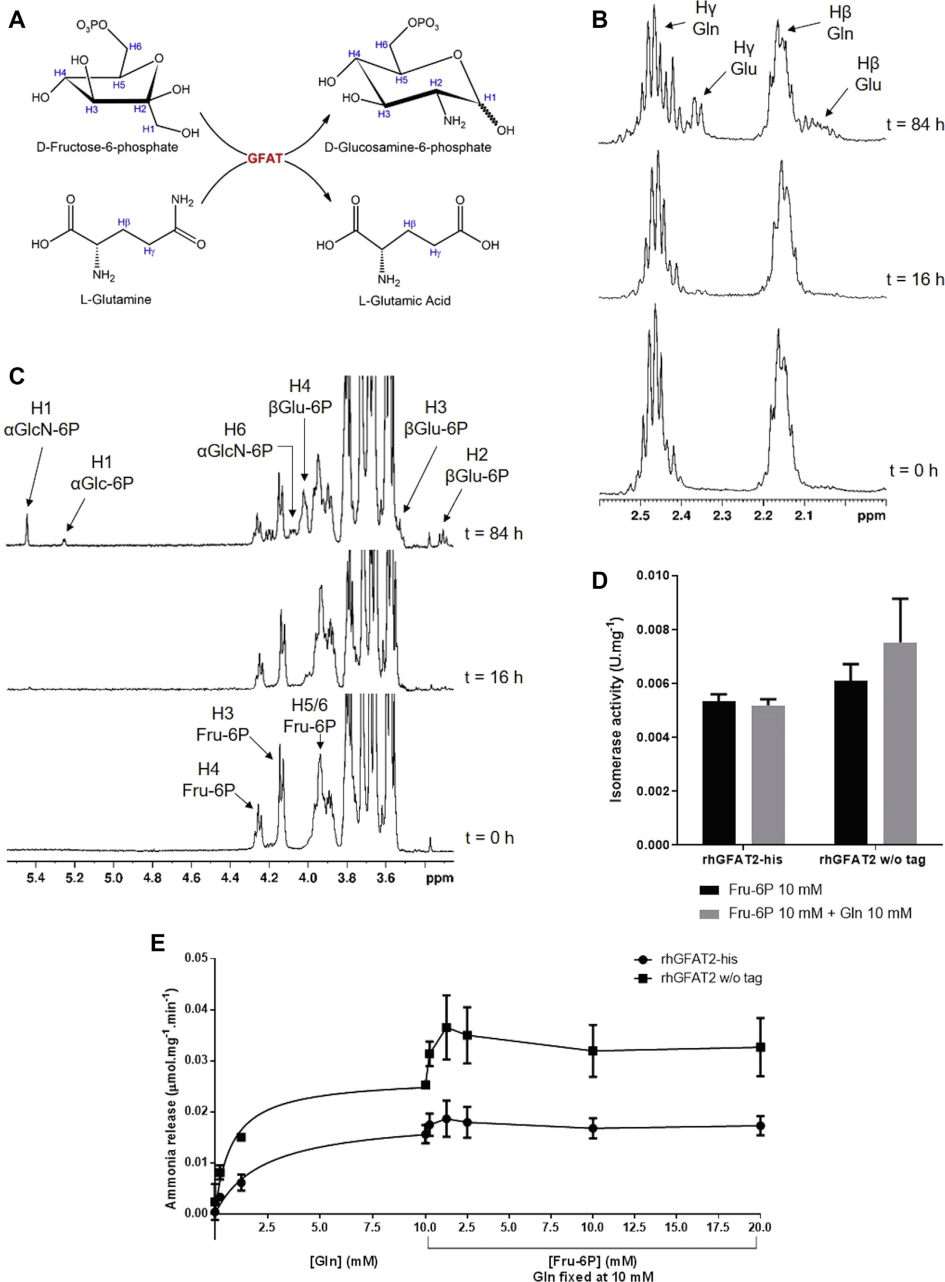
**Exploring PGI-like activity of rhGFAT2.***A*, reaction scheme of complete GFAT reaction. *B*–*C*, time course of rhGFAT2-his reaction in presence of Gln and Fru-6P (both at 3 mM) in 50 mM deuterated phosphate buffer pH 7.4 with 1 mM DTT at 25 °C, monitored by ^1^H NMR spectroscopy. The times at which spectral data were acquired refer to the addition of rhGFAT2-his (100 μg) as t = 0 h. *B*, region of ^1^H NMR spectra detailing Gln and Glu peaks. *C*, region of ^1^H NMR spectra detailing Fru-6P, Glc-6P, and GlcN-6P peaks. *D*, isomerization of Fru-6P in Glc-6P catalyzed by rhGFAT2-his or rhGFAT2 without (w/o) HisTag assessed either in the absence (*black bars*) or in presence (*gray bars*) of Gln. *E*, ammonia release from Gln, catalyzed by rhGFAT2-his (*black circles*) and rhGFAT2 without (w/o) HisTag (*black squares*). The enzyme was incubated with increasing amounts of Gln until 10 mM, and with fixed Gln at 10 mM and variable concentrations of Fru-6P as indicated.

To confirm the unproductive hydrolysis of Gln, we used nuclear magnetic resonance (NMR) to directly monitor the complete rhGFAT2-his activity (Fig. 2*A*). 1D ^1^H spectra were acquired by incubating equimolar amounts (3 mM) of Gln and Fru-6P in the presence or absence of rhGFAT2-his (Fig. S4, *A*–*B*). As expected, we observed the consumption of both Gln (reduced peaks at 2.15 and 2.48 ppm, corresponding to Hβ and Hγ, Fig. 2*B*) and Fru-6P (reduced peaks at 4.25 and 4.17 ppm, corresponding to H1 and H3, Fig. 2*C*) concomitantly with the generation of Glu (increased peaks at 2.07 and 2.36 ppm, corresponding to Hβ and Hγ, Fig. 2*B*) and αGlcN-6P (increased peaks at 5.42 and 4.062 ppm, corresponding to H1 and H6, Fig. 2*C*). We also noticed that peaks from both Glc-6P anomers increased during the reaction time course (5.23, 3.28, 3.52, and 4.00 ppm corresponding to αH1, βH2, βH3, and βH4, respectively, Fig. 2*C*). The H1 from β-sugars was not detected, probably due to distortion of the spectra by the water suppression at 4.70 ppm. However, TOCSY spectrum at t = 84 h exhibits the correlation signals among H1, H2, and H3 from βGlc-6P (4.65, 3.28, and 3.52 ppm, respectively, Fig. S4*C*). The TOCSY spectra also exhibit the correlation signals among Hα, Hβ, and Hγ from both Gln and Glu (Fig. S4*C*). Although close to the noise, the correlation signals between H1 and H3 from αGlcN-6P (5.42 and 3.93 ppm, respectively) and among H1, H3, and H5 from αGlc-6P (5.23, 3.75 and 3.92 ppm, respectively) were also observed (Fig. S4*C*, insert). The αH1 signal from Glc-6P is present in a proportion of 1:2.5 relative to αH1 of GlcN-6P measured in 1D ^1^H spectra, showing that rhGFAT2 partially acts as an isomerase even at equimolar concentrations of both substrates, corroborating the isomerase kinetics data. We did not observe spontaneous isomerization from Fru-6P to Glc-6P, spontaneous hydrolysis of Gln or GlcN-6P formation in the absence of the enzyme (Fig. S4*B*), and the oxidation of DTT (32) was the only alteration in 1D ^1^H spectrum observed in the absence of the enzyme (Fig. S4*B*).

To evaluate whether the lack of an effect of Gln on isomerase activity (Fig. S2*C*) was due the presence of the C-terminal HisTag, we performed the assay with rhGFAT2 without the tag. As observed for rhGFAT2-his, the addition of Gln did not reduce the ISOM activity of rhGFAT2 w/o tag (Fig. 2*D*), suggesting that part of the ammonium released from Gln hydrolysis in GLN domain does not reach the ISOM domain.

We further assessed the ammonia release from glutamine hydrolysis using a coupled assay with glutamic acid dehydrogenase in the presence of α-ketoglutaric acid and NADH. The reduction in NADH absorbance correlates to ammonia release and a standard curve of NH_4_Cl is used for quantification. As shown in Figure 2*E*, the release of ammonia increased as Gln concentrations increase for both the rhGFAT2 with and without HisTag. Michaelis–Menten equation fitting indicated that both enzymes reached a plateau at Gln saturating concentration (10 mM), but the ammonia leakage observed for rhGFAT2 w/o tag was twice the values observed for the HisTaggeg enzyme. Furthermore, the addition of Fru-6P, even at high concentrations, did not abolish the release of ammonia to the medium for both the enzymes (Fig. 2*E*), but actually enhanced the ammonia release, mainly for rhGFAT2 w/o tag. These data indicate that a great amount of the ammonia hydrolyzed from Gln is lost to the medium instead of reaching the ISOM domain for generation of GlcN-6P.

### rhGFAT2 inhibition by UDP-GlcNAc

UDP-GlcNAc, the final product of HBP, has been described as a potent inhibitor of glutaminase activity of hGFAT1 (20, 22). To examine whether UDP-GlcNAc is able to inhibit hGFAT2 as well, we assessed the glutaminase activity of rhGFAT2-his in the presence of different concentrations of the activated monosaccharide. By plotting the results in a Dixon plot (Fig. S5), we observed that UDP-GlcNAc is able to inhibit only 10% of rhGFAT2-his activity, behaving as a partial inhibitor.

### Unstructured loop as a key for interdomain (miss)communication

**Figure 3:**
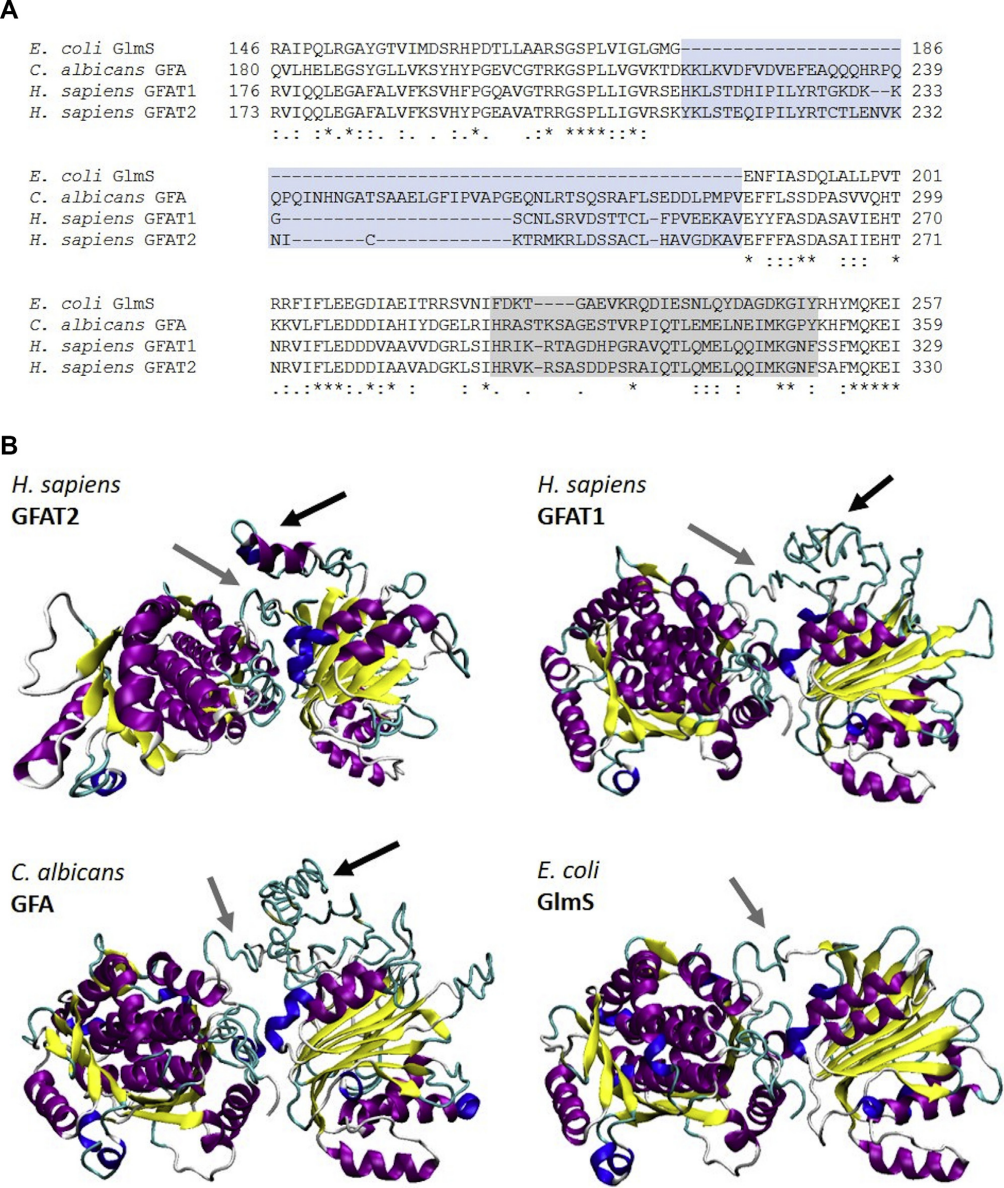
**Structure insights on human, fungal, and bacterial GFATs.***A*, alignment of the internal loop and interdomain connection sequences (highlighted in *blue* and *gray*, respectively) from GlmS (*E. coli*), GFA (*C. albicans*), and GFAT1 and GFAT2 (*H. sapiens*) performed with Clustal Omega server. *Dashes* indicate gaps, *asteriscs* indicate identical residues, and *dots* indicate residues with similar physical–chemical properties. *B*, three-dimensional models obtained for hGFAT2, hGFAT1, and GFA from *C. albicans* from threading using I-TASSER server. The structure of GlmS was retrieved from PDB under ID 4AMV. The proteins are represented in cartoon and colored according their secondary structure (α-helix in *purple*, β-sheet in *yellow*, 3–10 helix in *blue*, turns in *cyan*, and coil in *white*). The *black arrows* indicate the loop regions; the *gray arrow*s indicate the interdomain region.

In an effort to understand the differences between the kinetics data reported for other GFATs and our results, we compared the sequences of GlmS (GFAT from *E. coli*), GFA (GFAT from *Candida albicans*), hGFAT1, and hGFAT2. The alignment between GlmS and the hGFATs showed that the human variants exhibit an additional 46 residues in their sequences, from Lys211 to Val256 in case of hGFAT2 (Fig. 3*A*). This internal sequence is also present in GFA but is longer than those from hGFATs (Fig. 3*A*). Besides, these internal sequences are the most variable region among hGFATs and GFA and even between hGFAT1 and hGFAT2 (Fig. 3*A*).

To evaluate the impact of these internal sequences on GFATs’ structures, we generated tridimensional models for hGFAT2 using threading methods by subjecting the hGFAT2 amino acid sequence to I-TASSER and LOMETS servers. We opted for the I-TASSER final model because it had the best loop conformation. In this model, part of the loop (black arrow) was in α-helix conformation due to the interaction with intrachain residues and the stabilization occurred by interaction with the interdomain connective portion (gray arrow, Fig. 3*B*). The model reliability was corroborated by circular dichroism results obtained from rhGFAT2-his analysis, which indicated similar content of secondary structure (Fig. S6, Table 3).

**Table 3:** Secondary structure composition of rhGFAT2, based on experimental and theoretical data

Method	α-Helix	β-Sheet	Random
Circular dichroism^a^	33%	18%	49%
Molecular dynamics^b^	36%	19%	45%

a The secondary structure content of rhGFAT2-his was estimated from circular dichroism data, using different algorithms available on the Dichroweb server.

b The average secondary structure of GFAT2 from molecular dynamics simulation time was shown.

We also constructed tridimensional models for hGFAT1 and GFA from *C. albicans* by the same approach used for hGFAT2. As shown in Figure 3*B*, the additional sequence of hGFAT1 also formed an unstructured loop, similar to that observed for hGFAT2, which is also close to the interdomain region. In GFA, this region is bigger and even less structured, but is also next to interdomain connective portion, contrasting therefore to the GlmS structure (PDB ID: 4AMV), in which such a loop is absent (Fig. 3*B*).

**Figure 4:**
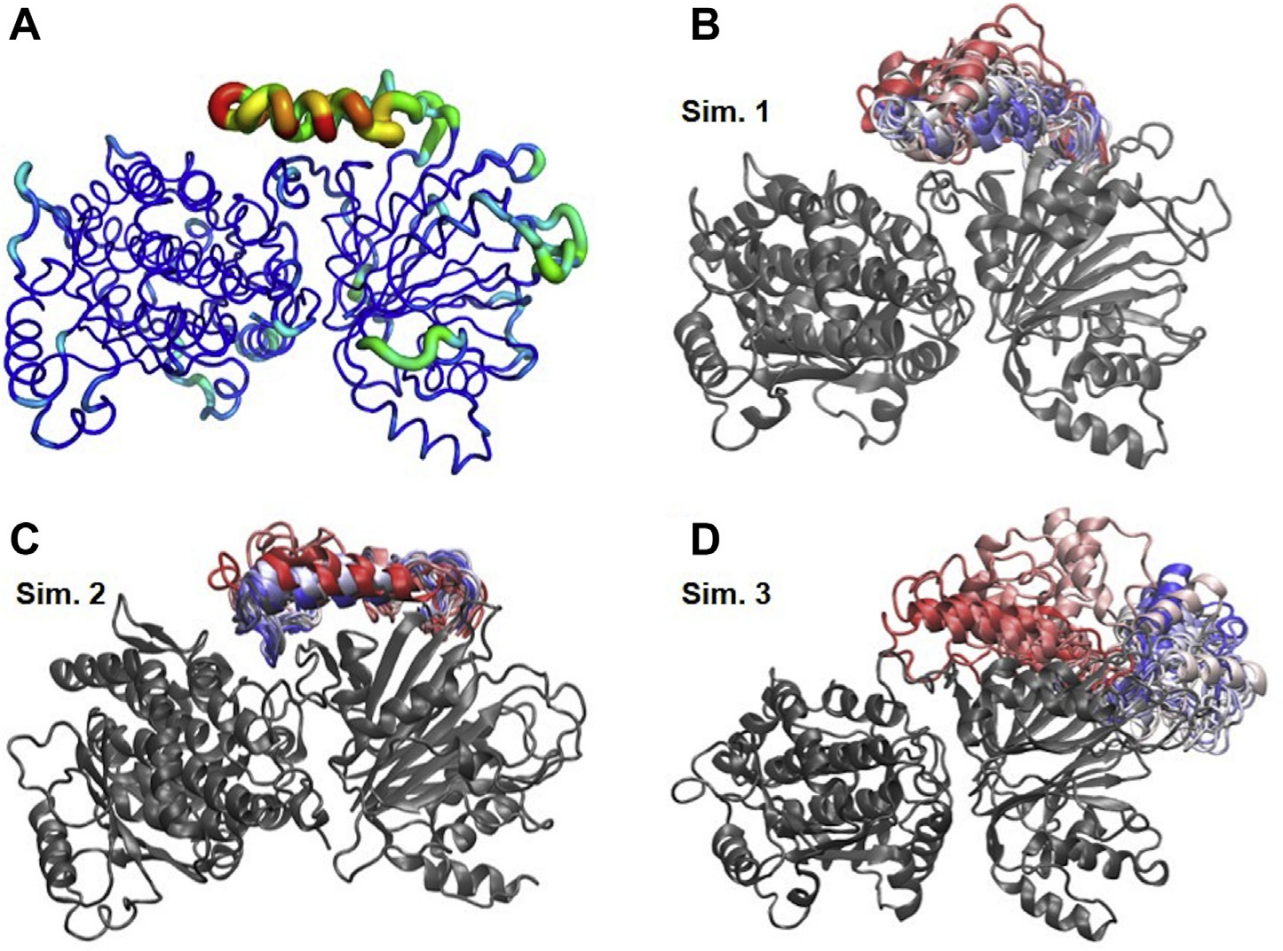
**Loop stability.***A*, three-dimensional root mean square fluctuation (RMSF) of protein residues during MD simulation 1. The protein is shown in tubes, whose thickness and color reflect the extent of each residue fluctuation (0.8–7.4 Å). *B*–*D*, final frames from MD simulations, with the protein represented in cartoon and colored in *gray*. The loop conformation is shown throughout simulation time and colored accordingly: initial frames are colored in *red*, intermediates in *white*, and final frames in *blue*.

**Figure 5:**
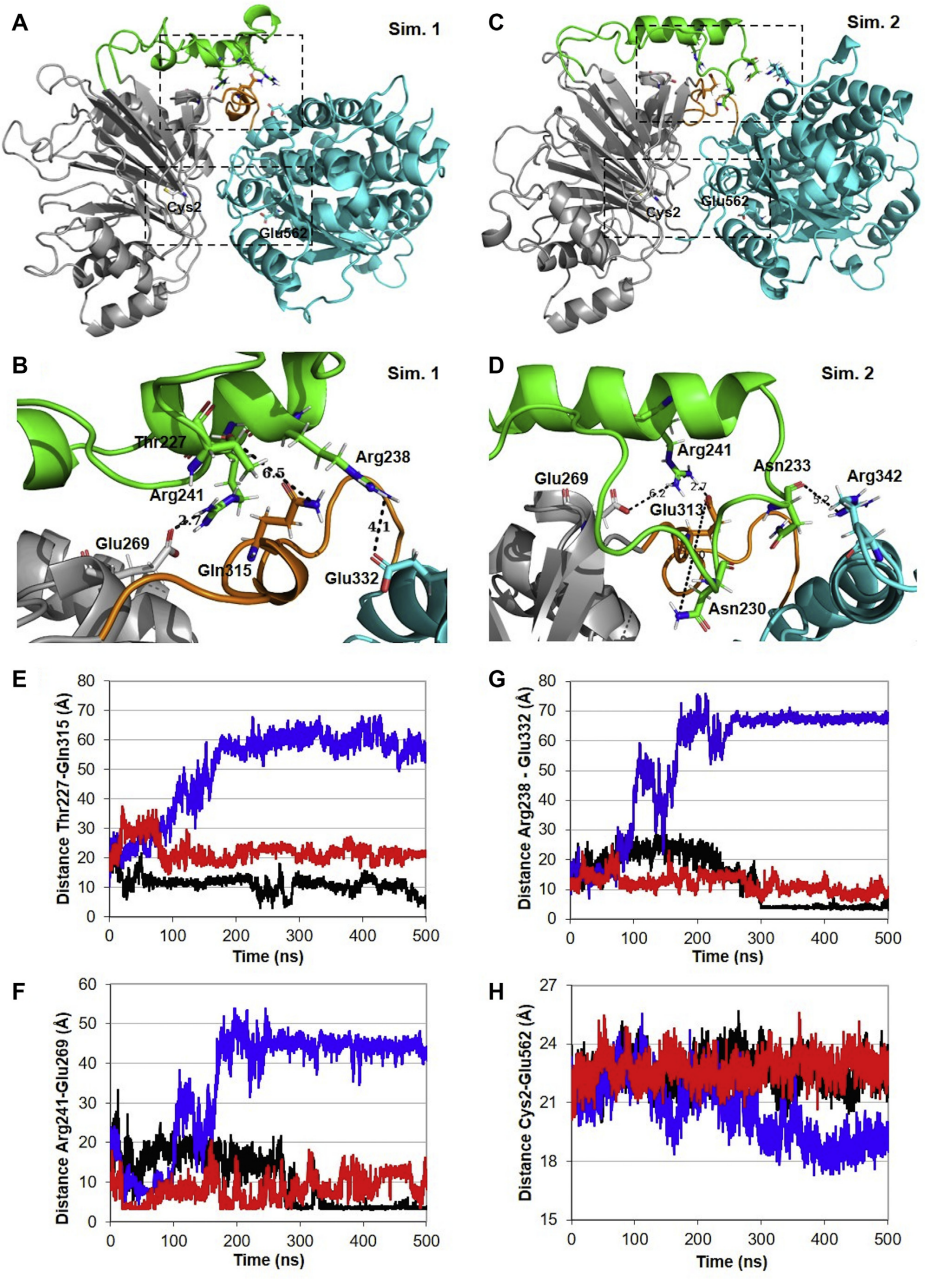
**Structural features of rhGFAT2.***A*–*D*, views of rhGFAT2 from the most populated cluster from MD simulations 1 and 2, depicting the full protein structure (*A* and *C*) and closer views of the loop region (*B* and *D*). The protein structure is represented in cartoon and its regions are colored as follows: the GLN domain in *gray*, the loop in *green*, the ISOM domain in *cyan*, and the interdomain region in *orange*. Key residues monitored in distance analysis are represented as sticks and the distances are represented by *dashed black traces*. *E*–*G*, analysis of the distances between the residues Thr227 and Gln315 (*E*), Glu269 and Arg241 (*F*), and Arg238 and Glu332 (*G*), reflecting the interaction of the loop with the interdomain region, the GLN domain, and ISOM domain, respectively, throughout simulation time. *H*, distance between catalytic residues from GLN domain (Cys2) and ISOM domain (Glu562) during MD simulation time. *Black lines* represent the data from simulation 1, *red lines* from simulation 2, and *blue lines* from simulation 3.

These results prompted us to investigate a possible function for the loop. Thus, we performed 3-replica of molecular dynamics (MD) simulations of 500 ns each using the AMBER package. In fact, the loop was the most flexible region of hGFAT2 structure, as shown by root mean square fluctuation analysis (RMSF, Fig. 4*A*). During the simulation time, we observed that the loop approached the protein in two simulations (Fig. 4, *B*–*C*), but moved away in the third simulation (Fig. 4*D*). We noticed that Thr227, Asn230, Asn233, Arg238, and Arg241 are major players for the loop interaction with the interdomain region (mainly through residues Glu313 and Gln315, Fig. 5, *A*–*E* and Fig. S7*A*). Those residues were also responsible for the interaction of the loop with GLN domain (through Glu269, Fig. 5, *A*–*D* and F) and ISOM domain (through the residues Arg342 and Glu332, Fig. 5, *A*–*D*, G and Fig. S7*B*).

Cluster analysis of MD frames from simulations 1 and 2 showed a heterogeneous population distribution, in which few clusters—the ones reporting the loop in close contact with interdomain region and ISOM domain residues—accounted for more than half of the frames (Fig. S7*D*), while the same analysis of simulation 3 produced a greater number of less populated clusters (Fig. S7*D*). These results indicate that the interaction between the loop and protein residues ensures its stabilization.

To assess whether the loop dynamics affects the movement of the domains, we monitored the distance between key residues from catalytic sites of both domains—Cys2, the suggested N-terminal nucleophile for glutamine hydrolysis on glutaminanse domain, and Lys559 and Glu562 (equivalent to Lys485 and Glu488 from GlmS) from ISOM domain. We observed that the GLN and ISOM domains did not move substantially in simulations 1 and 2, but in simulation 3 they get closer by 4 to 5 Å (Fig. 5, *A*–*D* and H and Fig. S7*C*).

**Figure 6:**
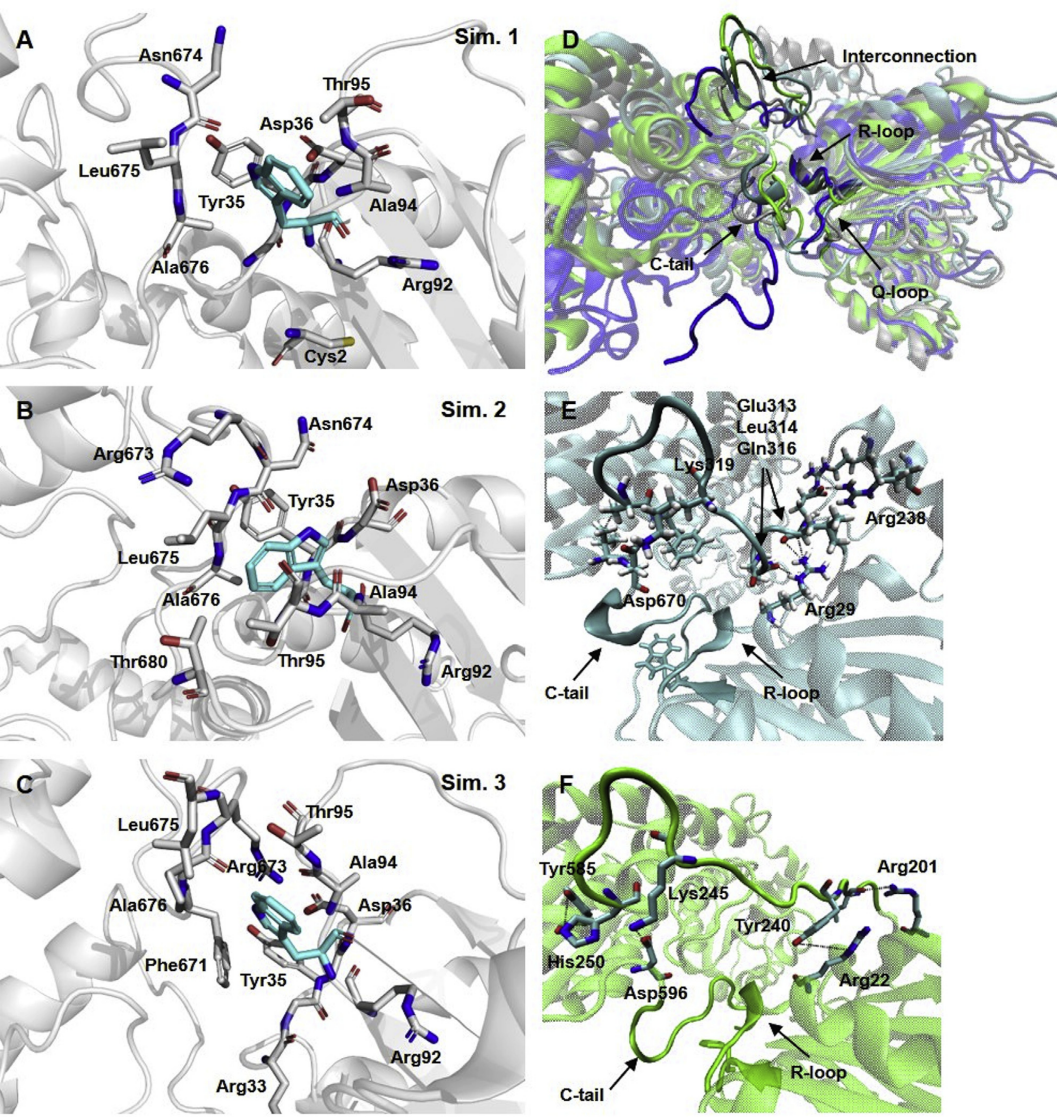
**Tryptophan neighborhood in hGFAT2 and GlmS.***A*–*C*, closer view of the residues that are 4 Å from Trp93 on the most populated cluster from MD simulations 1 (*A*), 2 (*B*), and 3 (*C*). The residues in contact to Trp93 are represented in sticks and colored in gray; Trp93 is also in sticks but colored in *cyan* (the hydrogens were removed for clarity). The protein backbone is represented in cartoon and colored in *gray*. *D*, views of the most populated cluster from MD simulations 1 (*gray*), 2 (*blue*), 3 (*cyan*), aligned to GlmS structure (*green*, PDB ID: 2J6H). The proteins were aligned by GLN domain and are represented in cartoon; the interdomain region, C-tail and Q- and R-loops are highlighted and indicated. *E*–*F*, closer view of the interdomain region in simulation 2 of hGFAT2 DM (*E*) and GlmS (*F*), depicting the interactions between this region and the overall protein. The residues involved in those interactions are represented in sticks and colored in *cyan*, and the distances are represented by *dashed black traces*.

We then evaluated the neighborhood of Trp93, the only Trp residue of this protein, equivalent to Trp74 in GlmS, to understand how the structure of hGFAT2 could affect the ammonia leakage. Even though we observed conserved interactions between Trp93 and residues from Q-, R-, and C-loops—such as Tyr35, Leu675, Ala676, and Arg33 (Fig. 6, *A*–*C*)—the C-tail is oriented upward to that observed in GlmS structure bound to DON and Glc-6P (Fig. 6*D*). In hGFAT2, this feature seems to be derived from the interaction between the loop and interdomain region and interdomain region with R-loop. Figure 6*E* shows that the hydrogen bonds between Arg29 and Glu313, Leu314 and Gln316 residues forced a turn in the interdomain connection. In contrast, Arg22 in GlmS, although close to Tyr240, seemed not to form a hydrogen bond to such residue, nor to any other within the interdomain region (Fig. 6*F*). Moreover, the sequence of interdomain region is distinct and contains three residues longer in hGFAT1 and hGFAT2 compared with GlmS (Fig. 3), which can linearly extend its structure up to 9.6 Å.

## Discussion

Despite the involvement of hGFAT2 in cancer aggressiveness, few studies to date have focused on the molecular and structural characterization of this protein. Here, we conducted a comprehensive study detailing the enzymatic properties of hGFAT2. We produced a recombinant hGFAT2 either fused or not to a HisTag at its C-terminal end and demonstrated that it is mostly found as tetramers, which is in agreement with previous data from hGFAT1 (21), but can also exist in higher-order oligomeric structures, possibly octamers, at lower extent. Even though the presence of higher-order oligomeric structures has not yet been reported for eukaryotic GFATs, the formation of multimeric forms could exert some regulatory role for hGFAT2, since the interplay between quaternary structures has been described for *E. coli* GlmS as a mechanism of enzyme inactivation (33).

Regarding the enzymatic properties of hGFAT2, the fitting of rate curves from GlcN-6P synthetic activity using the Michaelis–Menten equation resulted in ^*app*^*K*_*M*_ for Fru-6P and Gln similar to those described for mGFAT2 (24), hGFAT1 with no tags (23), and hGFAT1 His6-Asn298 (21, 22), but the ^*app*^*k*_*cat*_ were lower than those reported for mammalian and prokaryotic GFATs (20, 21). Interestingly, fitting the rate curves to ordered bisubstrate mechanistic model, based on previous kinetic studies with GlmS (34), resulted in an inconsistent value for ^*Fru-6P*^*K*_*M*_, suggesting that hGFAT2 does not follow such kinetic model. This perspective is corroborated by the aminohydrolyzing data, which shows that Gln is hydrolyzed by GFAT even in the absence of Fru-6P, whereas the addition of this monosaccharide-phosphate increases the glutaminase catalysis by fourfold. Such a feature was also observed for GFA from *C. albicans* (35). Taken together, these data suggest that Fru-6P binding is not essential for Gln binding, but acts as an activator of GFAT2 glutaminase activity. Since similar pattern of Fru-6P activation of aminohydrolyzing activity was observed to rhGFAT2 w/o HisTag, we are confident that this phenomenon—the nonconditioning pattern of Fru-6P for Gln binding—is not an artifact derived from the HisTag. Although our data were not enough to determine the proper kinetic model that fits hGFAT2 GlcN-6P synthetic activity, it strongly indicates that this enzyme does not follow the ordered bi–bi substrate model, in contrast to the bacterial GlmS (19).

NMR spectroscopy was used to directly detect the progress of hGFAT2 catalysis. We found a considerable amount of Glc-6P, suggesting that hGFAT2 can partially act as an isomerase-only enzyme, regardless of Gln presence. This data is corroborated by the isomerase assays with both rhGFAT2 constructs. Despite the phosphoglucose isomerase (PGI)-like activity already being reported for GlmS (36) and for GFA in the absence of Gln (35), our data is the first data to describe that human GFAT also retains such a PGI-like activity, and moreover, that this activity is maintained even in the presence of Gln. Our findings are also in agreement with a recent report detecting Glc-6P upon cocrystallization of hGFAT1 with Gln and Fru-6P (22).

In the other hand, the isomerase data indicate that part of Gln is lost in an unproductive hydrolysis. Indeed, the ammonia release assays suggest that Fru-6P does not prevent the loss of NH_3_ to the medium. To measure the efficiency of ammonia transfer, Floquet *et al.* (37) used the ratio between the *k*_*cat*_ of synthase and hemisynthase (glutaminase) activities, which, in their work, is 84% for GlmS. We and Ruegenberg *et al.* (22) observed an efficiency of ammonia transfer of approximately 50 and 47% for hGFAT2 and hGFAT1, respectively, indicating that human GFATs, in fact, have a higher rate of ammonia leakage. Structural data from GlmS point to the formation of a hydrophobic channel formed among Trp74 in the Q-loop and C-tail residues upon the binding of the two substrates as a key event for avoiding ammonia leakage (31, 37, 38). The movement of the Q-loop upon substrates binding was also observed for hGFAT1 (22), but it did not prevent the low efficiency of ammonia transfer. In this regard, kinetic and mutagenesis data from GFA suggest that deletion of a sequence from GLN domain disrupts the communication of both domains and hampers the GlcN-6P synthesis, but retains their aminohydrolyzing and isomerase-only activities (35). In the present work, we observed that sequence from GFA is present in both hGFAT1 and hGFAT2 and folds in a highly flexible loop in hGFAT2. The flexibility of this loop was also reported for hGFAT1 (22) and may be the reason for the difficulty in getting crystals from eukaryotic GFATs, as noticed by Nakaishi *et al.* (39).

Our MD simulation data suggest that the loop alternates among what could be seen as conformational states, in which it interacts with the interdomain region and ISOM domain or shifts to an open conformation. These results are in line with GlmS data from MD simulations and normal mode analysis (40). The hinge movement, which is reported to be performed solely by the hinge connection residues in GlmS, may be modulated by the loop residues in hGFAT2 and possibly in hGFAT1. In addition, the hinge connection sequence is distinct and is three residues longer in both hGFAT1 and hGFAT2 compared with GlmS, which could alter the domains’ motions and affect the sealing of hydrophobic channel by changing the orientation of R- and Q-loops to C-tail. Thus, our data suggest that the loop evolved as an additional regulatory mechanism, which is corroborated by having conserved phosphorylation sites in GFA (35, 41), GFAT from *Drosophila melanogaster* (42), mGFAT2 (24), and hGFAT1 (43, 44, 45), a posttranslational modification that alters their enzymatic properties with direct impact in cell biology (46).

The differences among hGFAT1 and hGFAT2 go beyond the hinge connection and loop sequences or their catalytic efficiency: they also differ in susceptibility to allosteric inhibition by UDP-GlcNAc (20, 22). Our data is in accordance with previous work describing the partial inhibition of mGFAT2 by UDP-GlcNAc (24). The close interaction observed between the loop residues and Arg342, near the allosteric site, could lead to pocket hindrance and may explain the poor inhibition.

Despite these number of contrasting properties of hGFAT2 and hGFAT1, it is worth noting that both isoforms are simultaneously expressed in the vast majority of cell types, although their ratio varies among them (7, 18, 47). Moreover, several evidences have shown that their expression is modulated by different transcription factors (Xpb1s for hGFAT1 and NR4A1 for hGFAT2, for example) (47, 48) and is observed in distinct circumstances (18, 49, 50). This sheds light on the relevance of the difference between the characteristics of these two enzymes, suggesting that cells can take advantage of it by changing the ratio hGFAT1/hGFAT2. In addition, we cannot exclude the possibility that hGFAT1 and hGFAT2 form heterotetramers or other states of heterooligomers. Therefore, our work provides the first comprehensive set of data on the structure, kinetics, and mechanics of hGFAT2. Our results contribute to the knowledge of physiological roles and differences between GFAT isoforms. More studies addressing the interaction of hGFAT2 to substrates and ligands are important.

## Experimental procedures

### Construction of pET-hGFAT2 plasmids

The *gfpt2* gene was amplified from pCMV6-AC plasmid (Origene, USA) by PCR and inserted into bacterial expression plasmid pET23a (Novagen, USA) to construct either pET-hGFAT2 with and without HisTag. For both plasmids, the gene amplification was performed using the same sense primer 5’ GGAATTCCATATGTGCGGAATCTTTGCCTAC 3’ (containing a restriction site for Nde I), but distinct antisense primers: 5’ ATAAGAATGCGGCCGCTTCCACAGTTACAGACTTG 3’ for hGFAT2-His, and 5’ ATAAGAATGCGGCCGCTTATTCCACAGTTACAGACTTG 3’ for hGFAT2 without tag (both containing a restriction site for Not I), this last containing a stop codon right after the protein sequence. The reactions were performed as follows: 2 min at 94 °C followed by 35 cycles of 1 min at 94 °C, 1 min at 52 °C, and 2 min at 68 °C, with an extension step of 7 min at 68 °C. The amplified genes were then electrophoresed in 1% agarose gel followed by purification using PCR purification kit (Qiagen, USA). Both purified genes and plasmids were digested with Nde I and Not I prior to ligation using the T4 DNA ligase (New England Biolabs, UK). The recombinant plasmids pET-hGFAT2 (without tag) and pET-hGFAT2-his were inserted into electrocompetent *E. coli* DH5α cells and positive colonies were subjected to a PCR colony using the abovementioned primers. The reactions was performed as follows: 2 min at 94 °C followed by 35 cycles of 1 min at 94 °C, 1 min at 52 °C, and 2 min at 72 °C, with an extension step of 7 min at 72 °C. True positive clones were isolated and sequenced by using an ABI PRISM dye terminator cycle sequencing core kit (Applied Biosystems, USA).

### Expression of recombinant hGFAT2s (rhGFAT2s) in *E. coli*

Chemically competent *E. coli* Codon plus cells (Novagen, USA) were transformed with 200 ng of the pET-hGFAT2 (with or without tag) plasmids, and positive clones were selected in an LB-agar medium containing 100 μg/ml ampicillin and 34 μg/ml chloramphenicol at 37 °C overnight. A single positive colony was preinoculated in 10 ml of LB medium containing 100 μg/ml ampicillin and 34 μg/ml chloramphenicol, and this culture was stirred at 220 rpm at 37 °C overnight. The overnight culture was diluted to 1:50 in 1 l of fresh antibiotic-containing medium and grown at 37 °C until an optical density (O.D._600nm_) of approximately 0.7 to 0.8 was reached. The induction of protein expression was conducted with 0.5 mM IPTG followed by 6 h of expression at 25 °C with 220 rpm stirring. Thus, the cells were harvested by centrifugation at 5000*g* for 20 min at 4 °C, and the total-cell lysate was prepared.

### Purification of rhGFAT2s

The pellet was suspended in 25 ml of Buffer A (20 mM Tris-HCl pH 7.5, 500 mM NaCl, 1 mM DTT and 0.5% NP-40) in the presence of 1 mM PMSF and 0.5 μg/ml of each protease inhibitor: aprotinin, bestatin, pepstatin, and E-64 (Sigma Aldrich, USA). Then, 5 mg/ml of lysozyme, 10 μg/ml of DNase A, and 5 mM of magnesium chloride were added, and the solution was incubated for 30 min at 4 °C with stirring. The total-cell lysate was sonicated using ten cycles of 15 s on and 1 min off at 40% amplitude, followed by centrifugation at 37,200*g* for 20 min at 4 °C.

The supernatant fraction containing the rhGFAT2 protein (with or without tag) was subjected to purification using a Ni^+2^NTA affinity column (HisTrap HP 5 ml, GE Healthcare, USA). The column was equilibrated with ten column volumes (CV) of Buffer A prior to loading the sample at a flow of 1 ml/min. After this step, the nonspecific ligands were removed by washing the column with 5 CV of Buffer A. The elution was performed using a gradient of Buffer A and Buffer B (Buffer A with the addition of 0.5 M imidazole) at a flow rate of 2 ml/min. All collected samples were analyzed by SDS-PAGE, and the tubes containing the purified rhGFAT2-his were pooled and dialyzed against Storage buffer (20 mM Tris-HCl pH 7.5, 150 mM NaCl, 1 mM DTT, and 5% glycerol).

The purification of rhGFAT2 w/o tag required an additional step of purification with an anion exchange chromatography. The SDS-PAGE analyzed fractions from HisTrap column, which contained the rhGFAT2 w/o tag, were pooled and dialyzed overnight against buffer C (20 mM Tris-HCl pH 8.0, 1 mM DTT, 0.5% NP40) with 150 mM NaCl. The dialyzed protein was diluted in buffer C to reach 50 mM NaCl immediately before loading to a Q-sepharose HP column (5 ml, GE Healthcare, USA), previously equilibrated with 10 CV of Buffer C. The nonspecific ligands were removed by washing the column with 5 CV of Buffer C. The elution was performed using a gradient of Buffer C and Buffer D (Buffer C with the addition of 0.5 M NaCl) at a flow rate of 2 ml/min. All collected samples were analyzed by SDS-PAGE, and the tubes containing the purified rhGFAT2 w/o tag were pooled and dialyzed against Storage buffer.

The purity of rhGFAT2s was assessed by scanning the CBB-stained gels using ImageJ software (51). After background subtraction, the pixels corresponding to each rhGFAT2 band were divided by the sum of the pixels over the corresponding lane.

### Western blot

The purified proteins (2 μg each) were submitted to SDS-PAGE in 10% acrylamide gel and electrotransfered to nitrocellulose membrane. The membrane was blocked with 3% (w/v) bovine serum albumin in Tris-buffered saline with 0.1% (v/v) Tween-20 and incubated overnight at 4 ^o^C with anti-His (Santa Cruz Biotechnologies, USA). The membrane was then washed, incubated for 1 h under agitation with the secondary antibody (anti-mouse, Santa Cruz). After a second round of washing, the labeled membrane was developed with Femto ECL (Thermo Fisher Scientific) and exposed to ImageQuant LAS 500 (GE Healthcare). The membrane was stripped and labeled with anti-GFAT2 (Cell Signaling Technologies, USA) following the same procedure described above.

### Cross-linking assay

The purified rhGFAT2 protein (3, 5 and 10 μg) was incubated in PBS buffer in the presence or absence of 1 mM EGS for 30 min at room temperature. The reactions were stopped with addition of 30 mM of Tris-HCl pH 8.0. Approximately 10 μg of each sample was analyzed by a gradient SDS-PAGE assay (Bio-Rad, USA) followed by Coomassie Brilliant Blue staining.

### Size exclusion chromatography

The purified rhGFAT2-his protein was subjected to a size-exclusion chromatography using a Superdex 200 column (GE Healthcare, USA). The column was equilibrated with 1 CV of 20 mM Tris-HCl pH 7.5, 150 mM NaCl, 1 mM DTT, 0.5% NP-40 prior to sample loading at a flow rate of 1 ml/min. The fractions were collected and analyzed by SDS-PAGE. The molecular weight of rhGFAT2 oligomer was estimated according to the retention time of standard proteins (Thyroglobulin—669 kDa, Apoferritin—443 kDa, β-amilase—200 kDa, BSA—66 kDa, Carbonic anhydrase—29 kDa, and Citocrome C oxidase—12.4 kDa) acquired from Sigma Co.

### Characterization of rhGFAT2-his products by NMR

Solution of rhGFAT2-his was exchanged with deuterated sodium phosphate buffer (50 mM pH 7.4, with 150 mM NaCl and 1 mM DTT) using four cycles of dilution and concentration with Amicon Ultra 30K NMWL (Millipore, USA). Two-hundred microliters of 100 μg protein solution were incubated with 3 mM of Fru-6P and 3 mM of Gln in Shigemi tubes. In order to check for spontaneous product formation or substrate consumption, the same amounts of Gln and Fru-6P were incubated with 200 μl of the deuterated buffer in which the proteins were conditioned. NMR spectra were obtained at a probe temperature of 298 K on a Bruker Avance III 500 MHz equipped with a 5 mm self-shielded gradient triple resonance probe. The GFAT reaction products were monitored by unidimensional ^1^H spectra, performed according to the Bruker pulse sequence zgesgp. The product characterization was assisted by total correlation spectroscopy (TOCSY) spectra, which were recorded using mlevesgpph pulse sequence with a mixing time of 80 ms and 64 scans per t1 increment. For each scan, 8192 transients of 256 complex data points were acquired to a 10.0 ppm spectral width. The spectra were multiplied with a square cosine bell function in both dimensions and zero-filled twofold. The data acquisition and analysis were performed using spectrometer software Topspin 3.6 (Bruker Corporation).

### Enzyme assays

#### GlcN-6P synthetic activity

The specific GlcN-6P synthetic activity from rhGFAT2-his and rhGFAT2 w/o tag was assayed by incubating 100 μg of each protein with 10 mM Fru-6P, 10 mM Gln, 1 mM DTT in PBS pH 7.4 (100 μl of final reaction volume) for 1 h at 37 °C under agitation. The glucosamine-6-phosphate (GlcN-6P) formed in the reaction mixtures was determined as described by Queiroz *et al.* (26), based on Elson & Morgan (25). Briefly, 10 μl of 1.5% acetic anhydride (Sigma, USA) and 50 μl of 100 mM sodium tetraborate were added to the reaction mixture and incubated at room temperature for 5 min under agitation. The samples were then incubated at 80 °C for 25 min, cooled down at 4 °C for 5 min, and spun down for removing precipitated protein. The resultant acetylated GlcN-6P was derivatized with 130 μl of Ehrlich reagent in a 96-wells microplate incubated for 30 min at 37 °C and finally read at 585 nm in microplate reader (SpectraMax 190, Molecular Probes, USA). The absorbance of the samples not incubated with GFAT substrates was discounted and the concentration of GlcN-6P was determined comparing the resultant absorbance of the samples with GlcN-6P standards processed in the same manner. The specific activity was expressed as units (μmol of GlcN-6P synthesized per min at 37 °C) per mg of protein.

For kinetic analysis, the assay was performed as described, but with the following modifications: rhGFAT2-his was incubated with variable concentrations of one of the substrates (Fru-6P or Gln, at 0.156, 0.313, 0.625, 1.25, 2.0, and 2.5) while the other was fixed at saturating concentration (10 mM); the reaction mixtures were incubated by multiple time points up to 15 min, counted from the addition of the enzyme (time point 0 min was considered as the reaction mixture without the enzyme). At the end of incubation time, the reaction mixtures were processed for GlcN-6P derivatization as for the specific activity. For calculation of kinetic parameters, the progression curves were plotted, and the initial velocity was calculated (related to each substrate). The apparent kinetic parameters (*k*_*cat*_ and *K*_*M*_) were determined by direct fit of the rate *versus* substrate concentration data to the rate equation for Michaelis–Menten using GraphPad Prism version 8 (GraphPad, USA). The data were also submitted to fitting to both simple Michealis–Menten equation and ordered bisubstrate mechanistic equation using GraFit version 7 (Erithacus Software, USA).

#### Aminohydrolyzing (glutaminase) activity

GFAT glutaminase activity was determined using a coupled assay, based on Ye *et al.* (52). In the assay, the glutamate released by GFAT activity is oxidized by glutamic acid dehydrogenase (GDH) with concomitant 3-acetylpyridine adenine dinucleotide (APAD) reduction. The amidotransferase reaction was carried out in 200 μl of 20 mM phosphate buffer pH 7.4 with 50 μg of rhGFAT2-his, containing variable concentrations of Gln (0.156, 0.313, 0.625, 1.25, 2.5, 5.0, and 10.0 mM), and Fru-6P. APADH formation was monitored continuously by absorbance at 370 nm in Spectramax 190 instrument (Molecular Devices, CA, USA) for 1 h at 37 °C. The APADH concentration was derived from its molar extinction coefficient. Kinetic parameters were determined as described for GlcN-6P synthetic activity.

#### Isomerase activity

The isomerization of Fru-6P to Glu-6P by rhGFAT2 was assayed as described by Olchowy *et al.* (35). In brief, 50 μg of rhGFAT2-his was incubated with variable concentrations of Fru-6P in 200 μl of 50 mM Tris-HCl pH 7.4 with 1 mM DTT, 0.5 mM NADP (Sigma, USA), and 2.5 mU/μl glucose-6-phosphate dehydrogenase (G6PD from *Saccharomyces cerevisiae*, Sigma, USA). Some assays were performed in the presence of variable concentrations of Gln (0.5, 0.625, 1.25, 2.5, 5.0, and 10 mM) with fixed (0.625, 2.5, and 10 mM) concentrations of Fru-6P. NADPH formation was monitored continuously by absorbance at 340 nm in Spectramax 190 instrument (Molecular Devices, CA, USA) for 30 min at 25 °C. The NADPH concentration was derived from its molar extinction coefficient. Kinetic parameters were determined as described for GlcN-6P synthetic activity.

To evaluate the ISOM specific activity of rhGFAT2 with or without HisTag, each enzyme was incubated with 10 mM Fru-6P in the presence or absence of 10 mM Gln in the same conditions described above. The absorbance at 340 nm was read at the end of 30 min. The specific ISOM activity was expressed as units (μmol of NADPH synthesized per min at 37 °C) per mg of the enzyme.

#### Ammonia release

The release of ammonia from Gln hydrolysis catalyzed by rhGFAT2 was monitored by using the GDH in reverse direction, based on Floquet *et al.* (37). In this perspective, the ammonia released by GFAT activity is used in reductive amination of α-ketoglutaric acid (αKG), thereby with NADH oxidation. The assays were carried out by incubating 50 μg of rhGFAT2 (with or without HisTag) with variable concentrations of Gln (0.25, 1.25, and 10.0 mM), or with a fixed saturating concentration of Gln (10 mM) and variable concentrations of Fru-6P (0.25, 1.25, 2.5, 10, and 20 mM), in 200 μl of 20 mM phosphate buffer pH 7.4 with 1 mM DTT, 0.25 mM NADH, 2.5 mM αKG, and 30 mU/μl GDH. Reaction mixtures with the enzymes and the variable concentrations of Gln (0.25, 1.25, and 10.0 mM), but without αKG were used as blanks for their correspondent reactions. The NADH consumption was assessed by absorbance at 340 nm in Spectramax 190 instrument after 30 min incubation at 37 °C. The NADH consumed was taken as the difference between the final absorbance of each of the samples and their correspondent blanks without αKG. The ammonia released was determined comparing the resultant absorbance difference of the samples with the ones from NH_4_Cl standards. The results were expressed as unit μmol of ammonia per min per mg of protein.

### Peptide fingerprinting

Five micrograms of rhGFAT2-his was reduced with 3 mM DTT at 60 °C for 30 min and carbamidomethylated with 9 mM iodoacetamide at room temperature for 30 min in the dark. The protein was then digested with Trypsin Gold (Promega) 1:100 at 37 °C overnight in 10 mM ammonium bicarbonate pH 8.0, and the resultant peptides were cleaned up with POROS 20 R2 (Applied Biosystens). The sample was dried under vacuum, solubilized in 2% acetonitrile and 0.1% formic acid (FA) in water, and submitted to LC-MS in Nexera UPLC system (Nexera, Shimadzu, Japan) coupled to maXis Impact mass spectrometer (Q-TOF configuration, Bruker Daltonics) equipped with electrospray ionization source. Separation was accomplished in an Acquity CSH C18 UPLC column (150 m × 1 mm, 1.7 μm particle size, Waters) at 50 °C using a flow rate of 130 μl/min. After equilibration with 0.1% formic acid in water containing 2% acetonitrile, the peptides were injected and eluted using the following acetonitrile gradient: 2 to 8% in 2 min, 8 to 25% in 28 min, 25 to 50% in 10 min and kept at 50% for 2 min, 50 to 95% in 1.5 min and kept at 95% for 6 min. The electrospray source parameters were set as following: capillary voltage at 4.5 kV, end plate offset at −500 V, nebulizer gas at 1.2 bar, dry gas at 8 L/min, and dry temperature at 200 °C. Mass spectra were acquired in the positive-ion mode over the range *m/z* 50 to 1500 in data-dependent acquisition fragmentation mode at 1 Hz. The mass spectrometer was internally calibrated using 100 μM sodium formate solution.

The mass spectrometry data was processed using Mascot Search engine (Matrix Science) in BioTools software version 3.2 (Bruker Daltonics). The MS/MS data were searched against both the Uniprot Human amino acid sequence database and the hGFAT2 sequence, with and without Met1, for protein/peptide identification. The search was set up for full tryptic peptides with a maximum of 2 missed cleavage sites; carbamidomethyl cysteine and oxidized methionine were included as fixed and variable modifications, respectively. The precursor mass tolerance was set to 10 ppm, and the maximum fragment mass error was set to 0.05 Da.

### Circular dichroism

The circular dichroism (CD) experiments were conducted with hGFAT2-his in a Chirascan Circular Dichroism Spectropolarimeter (Applied Photophysics, UK) at 20 °C using a quartz cuvette with a 0.01 cm path length. Spectra from three scans from 260 to 190 nm at a 30 nm/min speed were averaged, and the buffer baselines were subtracted from their respective sample spectra. As a negative control, the protein was further denatured with 6 M guanidine-HCl and CD scans were repeated. The secondary structure content was estimated from fitting the far-UV CD spectra using the different algorithms, such as CDSSTR, K2D (53), and SELCON3 (54, 55), which is available on the Dichroweb server (56, 57).

### Modeling GFAT structures

Multiple sequence alignments of hGFAT2 and its orthologous enzymes were carried out using ClustalO (58). The hGFAT2 sequence was submitted to the I-TASSER (Iterative Threading Assembly Refinement) (59) server to achieve a complete structural model. The hGFAT1 and GFA (from *C. albicans*) sequences were submitted to I-TASSER server as well. The best models were selected based on the higher confidence scores and template modeling scores.

### Molecular dynamics simulation

The best hGFAT2 model was further submitted to molecular dynamics simulation to investigate its conformational stability. Molecular dynamics (MD) simulations were performed using the AMBER v. 14 software package (60) with the AMBER ff14SB force field (61). Explicit TIP3P water molecules (62) were used to solvate the hGFAT2 structure model in a cubic water box, using periodic boundary conditions. The protonation state of protein residues was assigned according to the values at pH 7.4 using the PROPKA software (63). The system was then neutralized by adding 1 Na^+^ ion to the simulation box. SHAKE algorithm (64) was applied to constrain all the bonds involving hydrogen atoms. Long-range electrostatic interactions were calculated with the PME method (65). The nonbonded interactions (Coulomb and van der Walls) were calculated using cutoffs of 8 Å.

The system was energy-minimized using 25,000 cycles of Steepest Descent algorithm followed by 25,000 cycles of Conjugated Gradient method with and without position restraint of 5 kcal mol^−1^ Å^−2^ for protein heavy atoms. The system was gradually heated from 0.15 to 300 K over 200 ps. Langevin thermostat (66) with a collision frequency of 0.067 ps^−1^ was used to control the temperature under a canonical ensemble and applying positional restrictions to the protein heavy atoms. Next, the pressure was applied until stabilized at 1 bar, using Berendsen barostat (67), by 7.5 ns under an isothermal and isobaric MD simulation with protein heavy atoms restrained to adjust the solvent density. The force constant for restraint was decreased gradually from 3 to 0 kcal^−1^ Å^−2^. Finally, 500 ns of production MD simulation with a time step of 2 fs was performed at a constant temperature of 300 K using Langevin themostat with a collision frequency of 5.0 ps^−1^ and a constant pressure of 1 bar controlled by Berendsen barostat (67) with a 1 ps pressure relaxation time. The MD trajectory was saved every 100 ps for analysis. The MD simulations were performed by using different seeds to generate initial velocities. The analysis and figures were made using PyMol (The PyMOL Molecular Graphics System, Version 1.2r3pre, Schrödinger, LLC.) and VMD (68) programs.

## Data availability

All the data relevant to the present work are contained within this article or available upon request to Isadora A. Oliveira (IBCCF/UFRJ, email: isadora@biof.ufrj.br).

## Conflict of interest

The authors declare no conflicts of interest regarding this article.
