# Supplementary material for: Enzymatic and structural properties of human glutamine:fructose-6-phosphate amidotransferase 2 (hGFAT2)
Source: J Biol Chem. 2020 Dec 17;296:100180. doi: 10.1074/jbc.RA120.015189 (PMC7948480; doi:10.1074/jbc.RA120.015189)
Supplement: Figures S1–S7 [file mmc1.pdf]

Supporting Information

Updates on enzymatic and structural properties of human glutamine: fructose-6-phosphate amidotransferase 2 (hGFAT2)

Isadora A. Oliveira[1*], Diego Allonso[1,2], Tácio V. A. Fernandes[3,4], Daniela M. S. Lucena[1], Gustavo T. Ventura[1], Wagner B. Dias[1], Ronaldo S. Mohana-Borges[5], Pedro G. Pascutti[3], Adriane R. Todeschini[1*].

This document contains: supplemental figures 1 to 7.

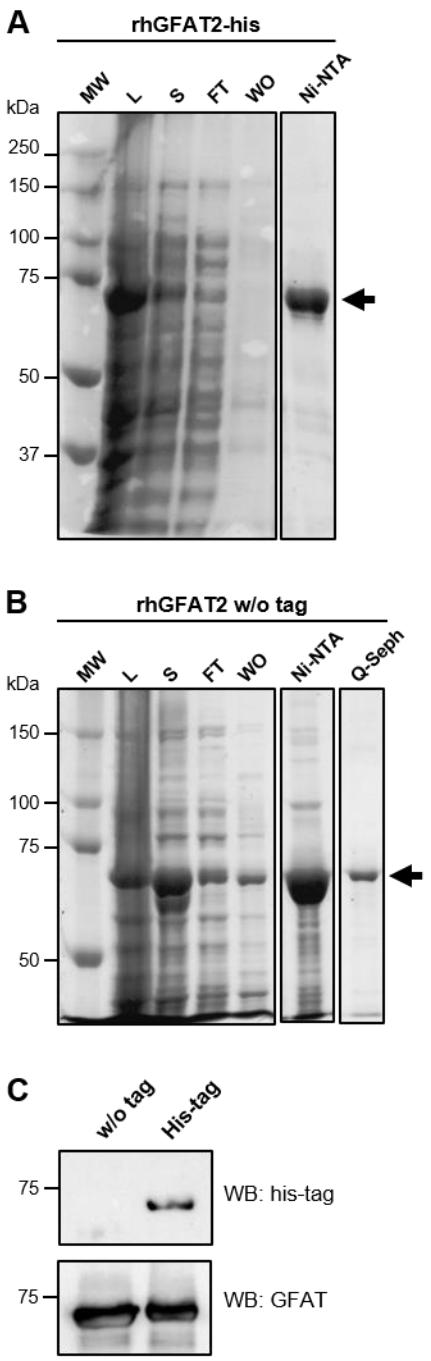

Suppl. Fig. 1 – Expression and purification of rhGFAT2 with or without a C-terminal HisTag. (A and B) SDS-PAGE of samples from purification steps of rhGFAT2 with (A) or without (w/o) a C-terminal HisTag (B) from *E. coli* cells. The lanes were loaded with *E. coli* cells' lysate (L), supernatant after centrifugation (S), flow through (FT) washout (WO) and eluate (Ni-NTA) from Ni^{+2}NTA affinity column (HisTrap). (B) For rhGFAT2 w/o tag, an additional step of anion exchange chromatography (Q-seph) was performed. The arrow represents a band of approximately 70 kDa, indicating the approximate molecular weight of rhGFAT2. The gels in (A) and (B) were stained with Coomassie blue G. (C) Western blot analysis of purified rhGFAT2s with and w/o tag, labeled with anti-HisTag and anti-GFAT antibodies. The molecular weight (MW) markers are indicated in each figure.

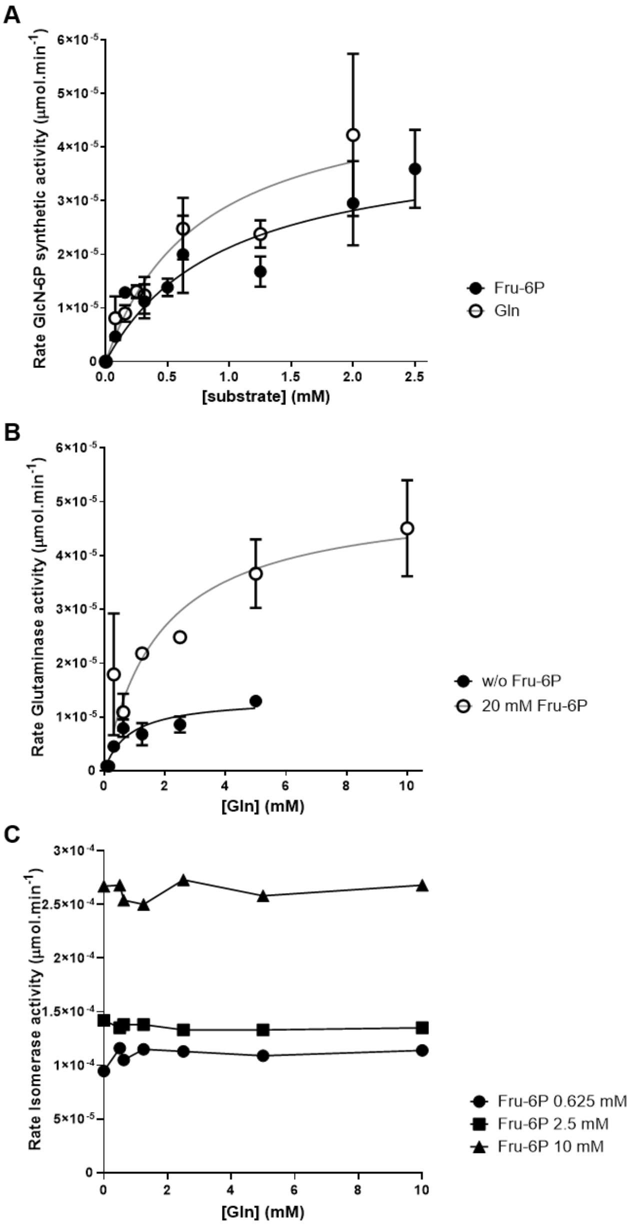

Suppl. Fig. 2 – Characterization of rhGFAT2-his enzyme activities' kinetics. Initial velocity *versus* substrate plots for GlcN-6P synthetic (A), glutaminase (B) and isomerase (C) activities of rhGFAT2-his. (A) hGFAT2-his was incubated with different concentrations of the indicated substrate (Gln, empty circles, or Fru-6P, black circles) while the other substrate was fixed in saturated concentration (10 mM). The GlcN-6P produced at each time point was detected and quantified using Elson-Morgan assay. The data was fitted to classical Michaelis-Menten model (solid lines). The calculated kinetic parameters are described in Table 1. (B) The hydrolysis of Gln from rhGFAT2-his glutaminase activity was detected using a coupled assay in order to quantify the Glu generated. To evaluate the influence of Fru-6P to rhGFAT2-his aminohydrolysing (glutaminase) activity, the enzyme was incubated with (empty circles) or without (black circles) saturated concentration (20 mM) of such phosphorylated monosaccharide, and in the presence of variable concentrations of Gln. The data was fitted to Michaelis-Menten model (solid lines). The calculated kinetic parameters are described in Table 1. (C) The impact of Gln in Fru-6P isomerization in Glc-6P by rhGFAT2-his was monitored using a coupled assay, with three fixed concentrations of Fru-6P (10 mM, black circles; 2.5 mM, black squares; and 0.625 mM, black triangles) and variable concentrations of Gln.

A

```
1   CGIFAYMNYR VPRTRKEIFE TLIKGLQRLE YRGYDSAGVA IDGNNHEVKE RHIQLVKKRG KVKALDEELY KQDSMDLKVE FETHFGIAHT RWATHGVPSA 100
101 VNSHPQRSDK GNEFVVIHNG IITNYKDLRK FLESKGYEFE SETDTETIAK LIKYVFDNRE TEDITFSTLV ERVIQQLEGA FALVFKSVHY PGEAVATRRG 200
201 SPLLIGVRSK YKLSTEQIPI LYRTCTLENV KNICKTRMKR LDSSACLHAV GDKAVEFFFA SDASAIIEHT NRVIFLEDDD IAAVADGKLS IHRVKRSASD 300
301 DPSRAIQTLQ MELQQIMKGN FSAFMQKEIF EQPESVFNTM RGRVNFETNT VLLGGLKDHL KEIRRCRRLI VIGCGTSYHA AVATRQVLEE LTELPVMVEL 400
401 ASDFLDRNTP VFRDDVCFFI SQSGETADTL LALRYCKDRG ALTVGVTNTV GSSISRETDC GVHINAGPEI GVASTKAYTS QFISLVMFGL MMSEDRISLQ 500
501 NRRQEIIRGL RSLPELIKEV LSLEEKIHDL ALELYTQRSL LVMGRGYNYA TCLEGALKIK EITYMHSEGI LAGELKHGPL ALIDKQMPVI MVIMKDPCFA 600
601 KCQNALQQVT ARQGRPIILC SKDDTESSKF AYKTIELPHT VDCLQGILSV IPLQLLSFHL AVLRGYDVDF PRNLAKSVTV EAAALEHHHH HH  692
```

B

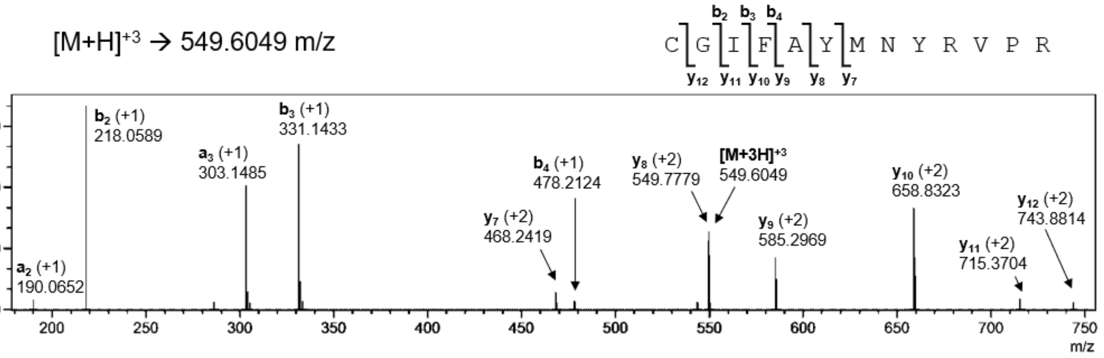

Suppl. Fig. 3 - Checking for removal of Met1 from hGFAT2-his. (A) rhGFAT2-his was digested with trypsin and the peptides was subjected to LC-MS/MS. The sequence coverage is highlighted in gray (62.6%). The N-terminal peptide sequence was assigned only upon the edition of the GFAT2 sequence for Met1 removal. (B) MS/MS spectrum of the N-terminal peptide ^{2}CGIFAYMNYRVPR^{14} (triply charged at *m/z* 549.6049), with assignment of *b* and *y* ion series (a_2 and a_3 ions were also observed) with their respective charge.

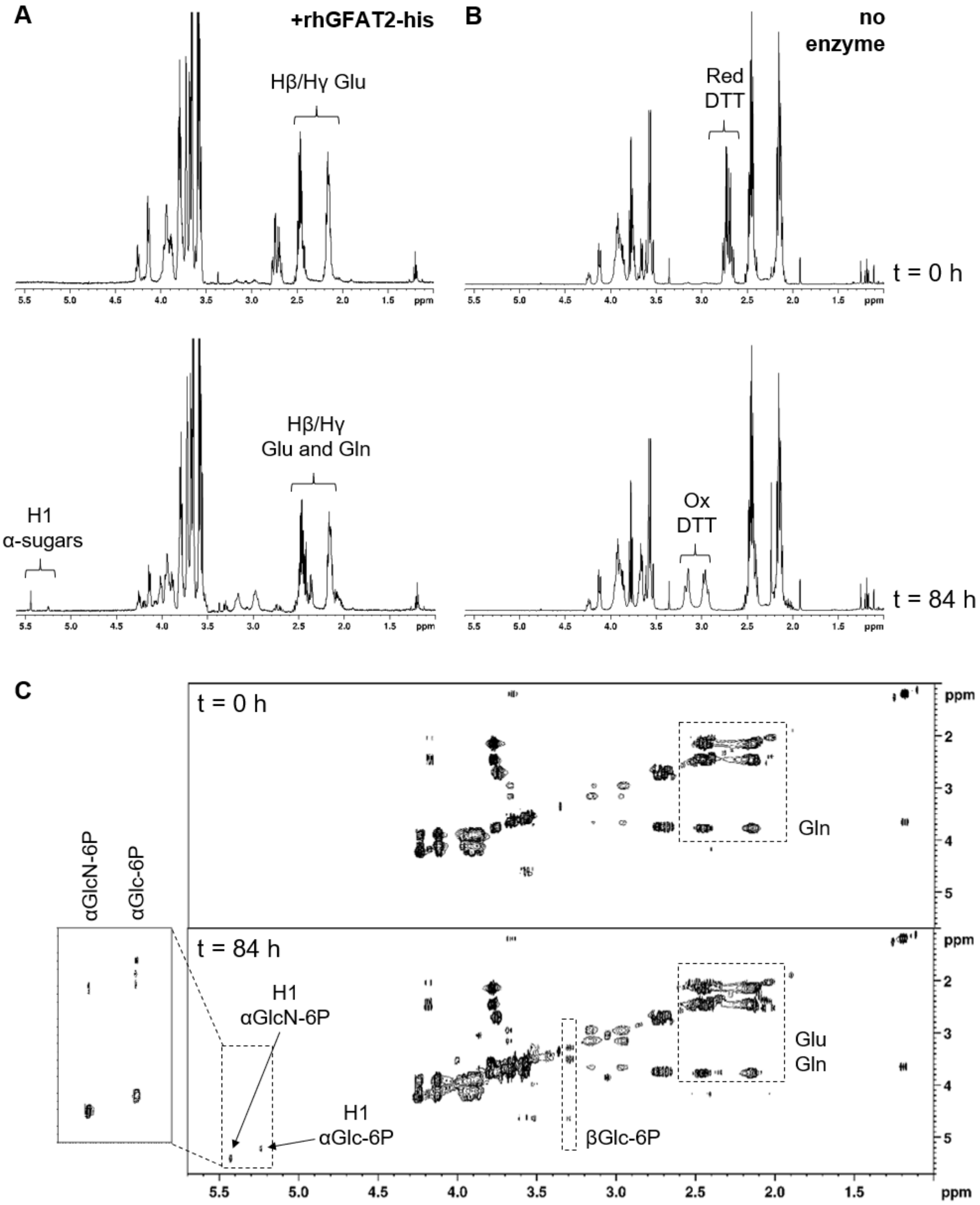

Suppl. Fig. 4 – Monitoring formation of GlcN-6P or Glc-6P by NMR. The spontaneous hydrolysis of Gln, isomerization of Fru-6P, or GlcN-6P formation were monitored for 84 h by 1D ^{1}H NMR in the same conditions of Fig. 2, but in the absence of rhGFAT2-his. The full spectra of the initial (t = 0 h) and final (t = 84 h) incubation time points of the reaction mix in the presence (A) or absence (B) of rhGFAT2-his are shown. The substrate's peaks are indicated, as well as the DTT's peaks of its reduced (t = 0) and oxidized forms (t = 84 h). (C) TOCSY spectra of initial (t = 0 h) and final (t = 84 h) reaction time points of the reaction mix with hGFAT2-his. The correlations involving ^{1}H protons from Gln, Glu, βGlc-6P, αGlc-6P and αGlcN-6P are indicated. The insert indicates the correlations among H1 and H3 from αGlcN-6P, and H1, H3, H5 and H6 from αGlc-6P observed at 84 h (the noise signal was removed for clarity).

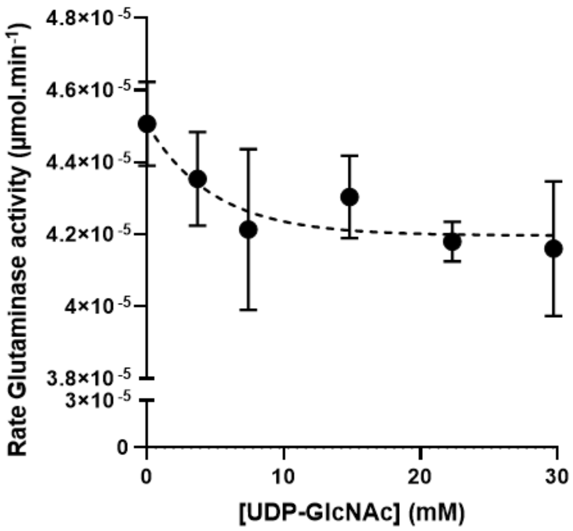

Suppl. Fig. 5 – Inhibition of rhGFAT2-his glutaminase activity by UDP-GlcNAc. Rate *versus* UDP-GlcNAc concentration plot of rhGFAT2-his. The data were fitted using one-phase decay and the plateau indicates 10% inhibition. Glutaminase activity assays were conducted under saturation concentration (10 mM) of both substrates Fru-6P and Gln.

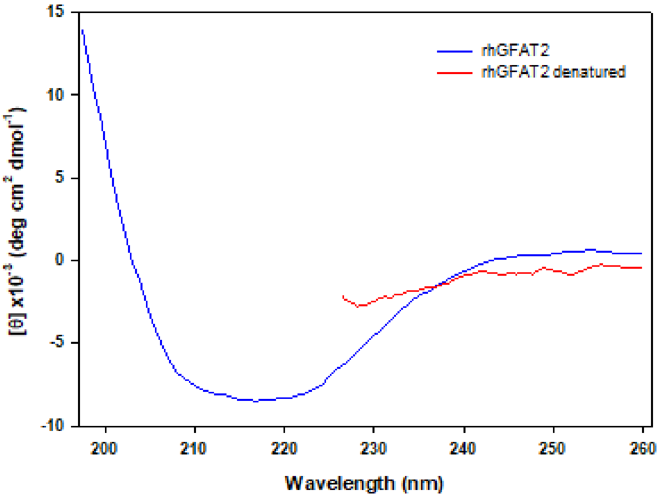

Suppl. Fig. 6 – Secondary structure composition of rhGFAT2. Circular dichroism spectra of rhGFAT2-his before and after denaturation with 6 M guanidine. Experiments were performed at 20 °C. Spectra were averaged from three scans at a 30 nm/min speed, and the buffer baselines were subtracted from their respective sample spectra.

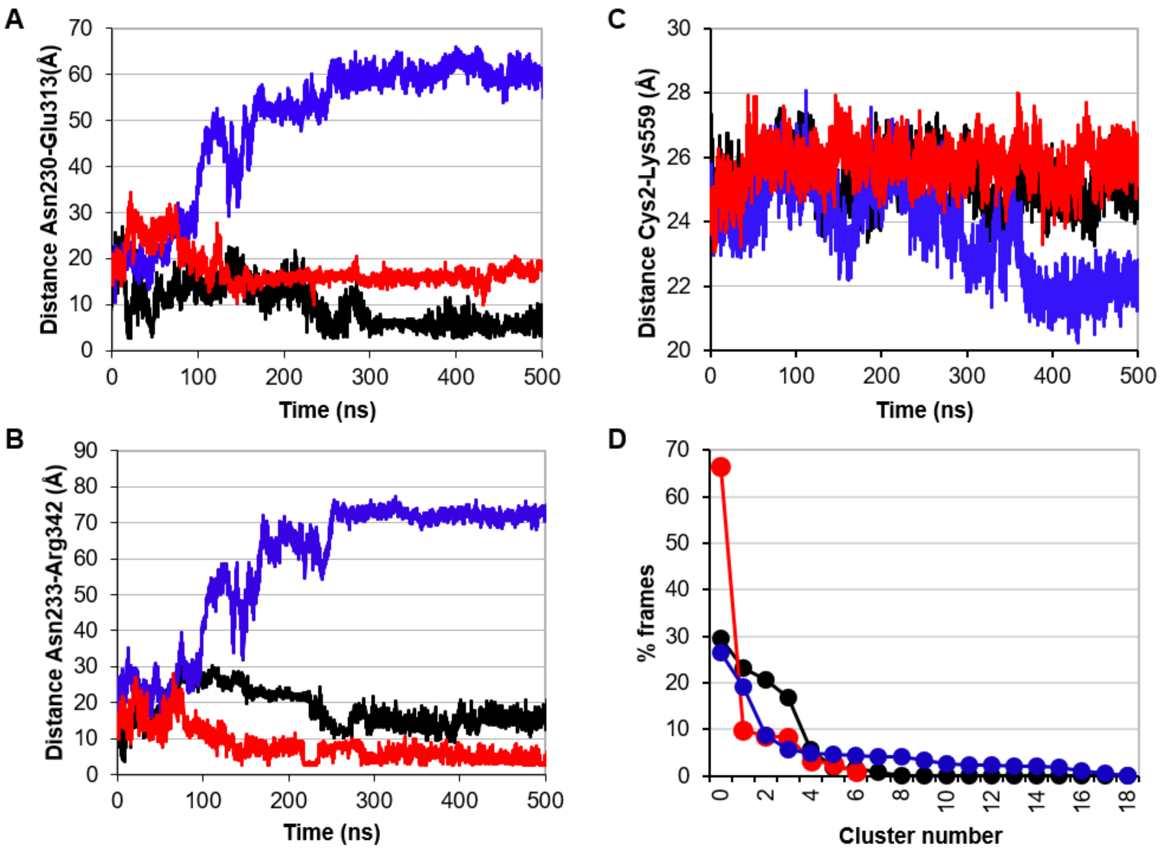

Suppl. Fig. 7 – Cluster and distance analysis (A-C) Distance analysis between Asn230 and Glu313 (A), Asn233 and Arg342 (B), and Cys2 and Lys559 (C). (D) Histogram of cluster analysis of the three MD simulations, plotting the percentage of frames from MD within each cluster. The cluster number correlates inversely with the simulation time. Black lines represent the data from simulation 1, red lines from simulation 2, and blue lines from simulation 3.
